# TRAF6 regulates melanoma invasion and metastasis through ubiquitination of Basigin

**DOI:** 10.18632/oncotarget.6886

**Published:** 2016-01-12

**Authors:** Zhongling Luo, Xu Zhang, Weiqi Zeng, Juan Su, Keda Yang, Lixia Lu, Chuan Bian Lim, Wen Tang, Lisha Wu, Shuang Zhao, Xuekun Jia, Cong Peng, Xiang Chen

**Affiliations:** ^1^ Department of Dermatology, Xiangya Hospital, Central South University, Changsha, Hunan, China; ^2^ Department of Pathology, Xiangya Hospital, Central South University, Changsha, Hunan, China; ^3^ Department of Genetics and Complex Diseases, Harvard T.H. Chan School of Public Health, Boston, MA, USA; ^4^ Department of Geriatrics, Xiangya Hospital, Central South University, Changsha, Hunan, China; ^5^ Institute of Medical Science Research, Xiangya Hospital, Central South University, Changsha, Hunan, China

**Keywords:** TRAF6, melanoma, invasion and metastasis, Basigin, ubiquitination

## Abstract

TRAF6 plays a crucial role in the regulation of the innate and adaptive immune responses. Although studies have shown that TRAF6 has oncogenic activity, the role of TRAF6 in melanoma is unclear. Here, we report that TRAF6 is overexpressed in primary as well as metastatic melanoma tumors and melanoma cell lines. Knockdown of TRAF6 with shRNA significantly suppressed malignant phenotypes including cell proliferation, anchorage-independent cell growth and metastasis *in vitro* and *in vivo*. Notably, we demonstrated that Basigin (BSG)/CD147, a critical molecule for cancer cell invasion and metastasis, is a novel interacting partner of TRAF6. Furthermore, depletion of TRAF6 by shRNA reduced the recruitment of BSG to the plasma membrane and K63-linked ubiquitination, in turn, which impaired BSG-dependent MMP9 induction. Taken together, our findings indicate that TRAF6 is involved in regulating melanoma invasion and metastasis, suggesting that TRAF6 may be a potential target for therapy or chemo-prevention in melanoma.

## INTRODUCTION

Melanoma, a type of skin cancer that originates from melanocytes, is one of the most deadly malignancies in the world. Melanoma often develops early metastasis and when the patient visits, the cancer has already formed metastasis. They have a poor prognosis, with a 5-year survival rate of less than 10% [1]. The annual incidence of melanoma is on the rise while the numbers for other tumors such as lung, prostate and cervical cancers are declining [2, 3], suggesting that melanoma remains a serious threat to public health. Although the causes of melanoma are not fully understood, the current consensus is that melanoma has genetic susceptibility in part and is a complex disease with genetic-environmental factors interaction.

Tumor necrosis factor Receptor-Associated Factors (TRAFs), a family of adaptor proteins, play important roles in signal transduction by interacting with the intracellular domains of many receptors [4]. Most TRAFs contain a highly-conserved N-terminal RING finger domain, variable numbers of zinc fingers and a C-terminal TRAF domain [5]. The TRAF domain is implicated in protein-protein interaction, while the RING and zinc finger domains mediate signaling events through its ubiquitin (Ub) E3 ligase activity [6, 7]. TRAF6 is recognized as a signal transducer, which activates the NF-κB pathway in response to pro-inflammatory cytokines. TRAF6 has E3 ligase activity and is responsible for inducing Lys-63 (K63)-linked poly-ubiquitination chains, functioning together with E2 Ubc13/Uev1A complex to mediate IKK activation [8–10].

Besides its roles in immunity and inflammatory response, TRAF6 has been documented as an oncogene in recent studies. The E3 ligase activity of TRAF6 is essential for K63 linked ubiquitination of Akt, which is required for membrane localization and oncogenic activation of Akt [11]. Amplification of TRAF6 was identified in lung cancer, which serves to activate NF-κB pathway in RAS-driven cancer [12]. Inhibition of TRAF6 suppresses NF-κB activation, and subsequent anchorage-independent growth and tumor formation [12]. In primary mouse bone marrow cells, overexpression of TRAF6 leads to a myelodysplastic syndrome that develops into a fatal acute myeloid leukemia [13]. It was also reported that TRAF6 increases HIF-1α expression and promotes tumor angiogenesis [14]. A recent study showed that TRAF6 overexpression was positively correlated with glioma grade and Ki-67 index, which in turn predicted poorer prognosis in glioma patients [15]. However, the role of TRAF6 in cancer invasion and metastasis, particularly in melanoma, is unclear.

CD147/Basigin (BSG), a member of the immunoglobulin superfamily, is an extracellular matrix metalloproteinases (MMPs) inducer (EMMPRIN). BSG is overexpressed in various tumors, including melanoma [16–18]. Our previous studies demonstrated that BSG can regulate melanoma invasion and metastasis through induction of MMPs and VEGF [16, 19].

In this study, we present data showing that TRAF6 is overexpressed in primary and metastatic melanoma tumors. Knockdown of TRAF6 significantly blocked melanoma cell invasion and metastasis *in vitro* and *in vivo*. Furthermore, TRAF6 can directly interact with and ubiquitinate BSG leading to MMP9 induction, which serves as a mechanism for melanoma invasion and metastasis.

## RESULTS

### TRAF6 is overexpressed in melanoma

**Figure 1:**
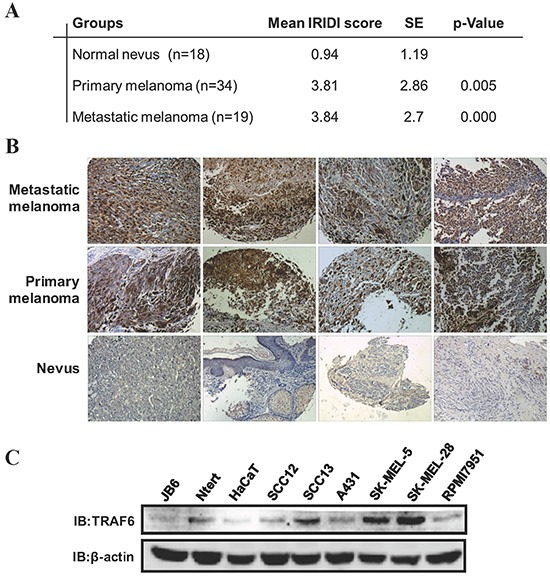
TRAF6 is highly expressed in melanoma **A.** A melanoma tissue array was stained using the TRAF6 antibody. IRIDI score was calculated and statistics analyzed as described in Materials and Methods. **B.** Representative images of immunohistochemical staining of TRAF6 in metastatic melanomas, primary melanomas and normal nevi were taken at 200× magnification. **C.** Immunoblot analysis was performed to examine TRAF6 expression in several normal and skin cancer cell lines by indicated antibodies.

To investigate the role of TRAF6 in melanoma, immunohistochemistry was performed on 18 nevi, 34 primary melanomas and 19 metastatic melanomas. As shown in Figure 1A and 1B, when compared with the nevus specimens, TRAF6 is overexpressed in both primary and metastatic melanoma tissues. We also examined TRAF6 expression in different skin cancer cell lines and found that TRAF6 is highly expressed in melanoma cell lines (SK-MEL-5 and SK-MEL-28), compared with other cell lines (Figure 1C), suggesting that TRAF6 might play an important role in melanoma.

### Knockdown of TRAF6 attenuates melanoma cell growth *in vitro* and *in vivo*

**Figure 2:**
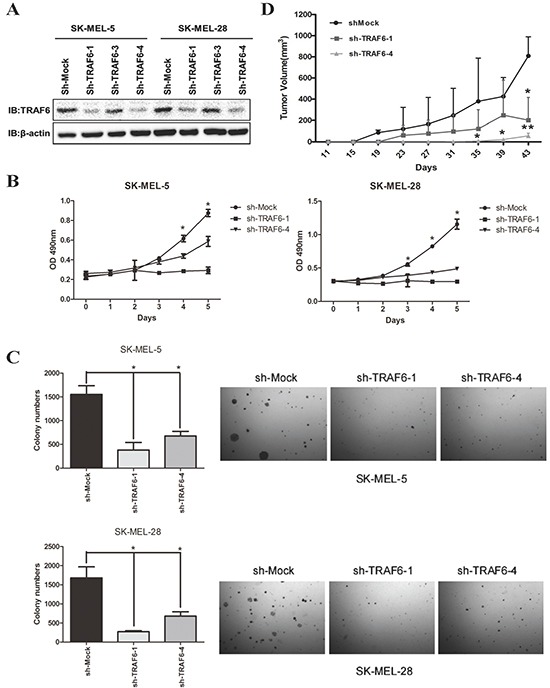
Downregulation of TRAF6 expression in human melanoma cells attenuates proliferation *in vitro* and *in vivo* **A.** Knockdown of TRAF6 was generated in SK-MEL-5 and SK-MEL-28 cell lines. TRAF6 protein expression was assessed by immunoblot (IB) analysis as indicated. **B.** shMock, shTRAF6#1 or #*4* SK-MEL-5 and SK-MEL-28 cells were seeded (1 × 10^3^ per well/100 μL) into 96-well plates and proliferation was assessed as described in *Materials and Methods*. Data from multiple experiments are expressed as means ± SEM. Significant differences were evaluated using a two-way ANOVA and the asterisk (*) indicates a significant difference (p < 0.05). **C.** SK-MEL-5 (upper panel) or SK-MEL-28 (lower panel) cells expressing shMock, shTRAF6 #1 or #4 were seeded in 0.3% BME agar containing 10% FBS. The cultures were maintained in a 37°C, 5% CO_2_ incubator for 10 days and then colonies were counted using a microscope and the Image J program. Data from multiple experiments were expressed as means ± SEM. Significant differences were evaluated using a one-way ANOVA and the respective significant differences are as indicated. **D.** SK-MEL-5 cells (5×10^6^/0.15mL) expressing shMock or sh-TRAF6 *(#1 and #4)* were injected into nude mice to establish subcutaneous xenografts. Once tumors were palpable, tumor volume was measured twice per week. Data represent means (n=5) ± SEM of each group. Bars, SD; *, P<0.05; **, P<0.01.

To study the effect of TRAF6 on melanoma cell growth, we generated stable knock down of TRAF6 by shRNA in SK-MEL-5 and SK-MEL-28 cell lines with independent shRNAs targeting sequences (Figure 2A). As shown in Figure 2A, shTRAF6 #1 and #4 showed the most significant knockdown at the protein level and thus were selected for subsequent experiments. The knock down of TRAF6 led to significant attenuation of proliferation and anchorage independent growth in both cell lines (Figure 2B and 2C). Furthermore, we conducted xenograft study in nude mice to examine the effect of knocking down TRAF6 on melanoma cell growth *in vivo*. Consistent with the *in vitro* data, knocking down TRAF6 led to significant delay in growth of xenografted melanoma tumors and resulted in smaller tumors compared with xenografts expressing scrambled shRNA (Figure 2D).

### Knockdown of TRAF6 blocks melanoma cell invasion and metastasis *in vitro* and *in vivo*

**Figure 3:**
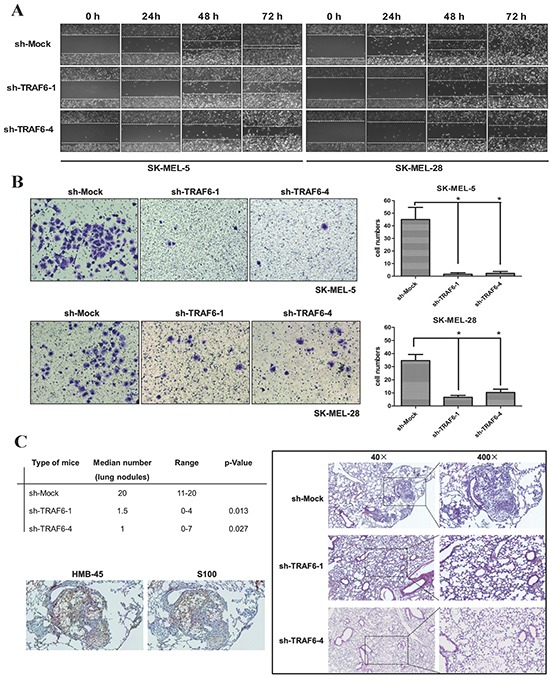
Knocking down TRAF6 inhibits melanoma invasiveness and metastasis *in vitro* and *in vivo* **A.** Wound healing assay was performed to examine the migration capability *in vitro* as described in *Materials and Methods*. Images (at 40x magnification) were taken every 24 h up to 72 h. **B.** Cells that migrated across the membrane were stained with crystal violetand imaged at 100x magnification. Data represent the means (n=3) ± SEM of each group. **C.** Average number of lung micro-metastasis per mouse from each group was determined. Representative microscopic H&E images of lung section were shown. Positive immunohistochemical images indicative of melanoma (HMB-45 and S100) were shown.

Given that TRAF6 is overexpressed in metastatic melanoma (Figure 1A), we hypothesized that TRAF6 might mediate melanoma invasion and metastasis. To directly test this hypothesis, we performed wound healing and transwell assays. We found that the number of migratory and invasive cells were significantly decreased in TRAF6-deficient SK-MEL-5 and SK-MEL-28 cells (Figure 3A and 3B). We next examined the effect of TRAF6 on metastasis *in vivo* using a lung metastasis mouse model. In agreement with the results *in vitro*, the size and number of metastatic nodules in lung were significantly reduced in TRAF6-knockdown cells (Figure 3C, left top). Immunohistochemical staining of lung nodules with HMB-45 and S100 (markers for melanoma) showed positive reaction (Figure 3C left bottom), which confirmed these cells originated from melanoma in lung.

### TRAF6 interacts with CD147

**Figure 4:**
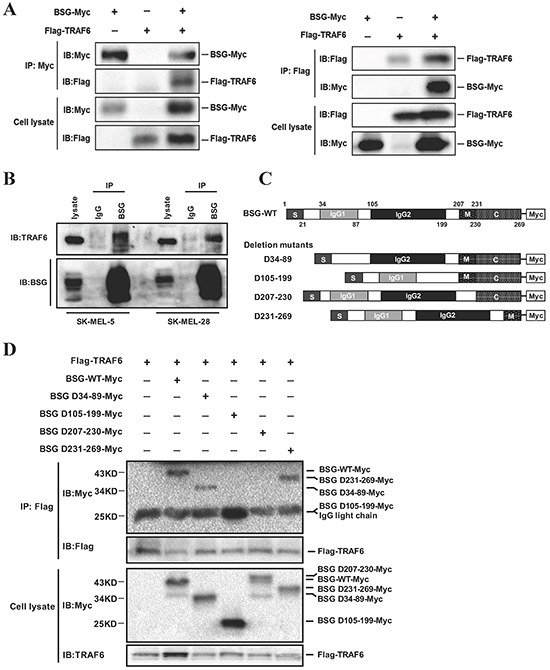
TRAF6 interacts with BSG **A.** TRAF6 binds to BSG. 293T cells were co-transfected with Flag-TRAF6 and BSG-Myc plasmids. Co-immunoprecipitation was performed with anti-Flag or anti-Myc, followed by immunoblotting with the indicated antibodies. **B.** TRAF6 binds to endogenous BSG. SK-MEL-5 and SK-MEL-28 cells lysates were immunoprecipitated using control IgG or anti-BSG antibody and the complex was detected by immunoblotting with anti-TRAF6. **C.** Schematic of the BSG/BSG mutant. **D.** The transmembrane domain mediates the interaction between BSG and TRAF6. Flag-TRAF6 and truncated BSG were co-transfected into 293T cells. At 36 h post-transfection, cell lysates were immunoprecipitated with anti-Flag antibody and then subjected to immunoblotting with anti-Myc or anti-Flag antibody.

BSG/CD147 has been shown to induce MMPs expression in stromal as well as in tumor cells [20, 21], which plays critical role in tumor invasion and metastasis. Notably, we determined that BSG is a novel interacting partner with TRAF6. The presence of BSG-Myc and Flag-TRAF6 in the immunoprecipitation complex was detected by anti-Myc and anti-Flag antibodies separately, after co-transfection in 293T cells (Figure 4A). The interaction between endogenous TRAF6 and BSG was also confirmed in both SK-MEL-5 and SK-MEL-28 cells (Figure 4B). To identify the domain of BSG responsible for binding to TRAF6, plasmids expressing Flag-TRAF6 together with full-length BSG or truncated BSG (Figure 4C) were transfected into 293T cells. We found that the transmembrane domain (D207-230) of BSG is required for TRAF6-BSG interaction (Figure 4D).

### TRAF6 regulates BSG ubiquitination and membrane recruitment

**Figure 5:**
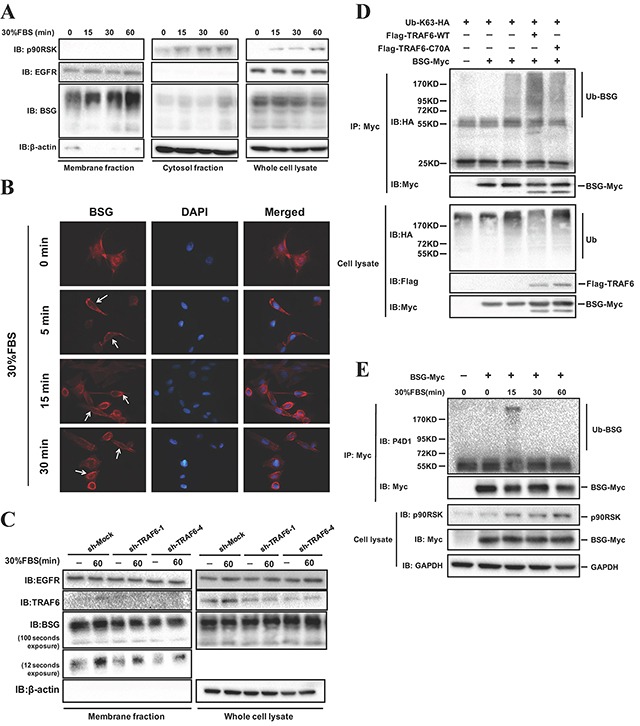
TRAF6 regulates BSG ubiquitination and plasma membrane recruitment **A.** BSG is recruited to the plasma membrane following FBS stimulation. SK-MEL-5 cells were starved for 16 h and treated with 30% FBS for the indicated time points. Membrane and cytosolic fractions were isolated as described in *Materials and Methods* and analyzed by immunoblotting with the indicated antibodies. **B.** SK-MEL-5 cells were serum starved, treated with 30% FBS for 5-30 min and fixed for immunofluorescence analysis. Nuclear DNA was stained with DAPI (blue). BSG subcellular translocation (red) was pointed by arrows. **C.** TRAF6 regulates the FBS-induced BSG plasma membrane recruitment. TRAF6-deficient SK-MEL-5 cells were starved for 16 h, and then treated with 30% FBS for indicated times. Membrane fraction extractions were examined by immunoblotting with indicated antibodies. **D.** TRAF6 is required for K63-mediated BSG polyubiquitination. 293T cells were co-transfected with Ub-K63-HA, along with TRAF6-WT-Flag or TRAF6-C70A-Flag and BSG-myc. At 36 h post-transfection, cell lysates were immunoprecipitated with anti-Myc. Ubiquitinated BSG was visualized by immunoblotting using anti-HA. **E.** FBS induces endogenous BSG ubiquitination. BSG-myc was transfected into SK-MEL-5 cells, at 24 h post-transfection, cells were starved for 16 h. After stimulation with 30% FBS, cell lysates were immunoprecipitated with anti-Myc. Endogenous ubiquitination of BSG was detected by P4D1 antibody.

Although BSG is assumed to be a membrane protein, it has been reported to be localized in the cytoplasm and mitochondria [22–25]. The dynamics and biological significance of BSG translocation remain unknown. Our results showed that BSG translocation to the membrane is dramatically increased upon serum stimulation in serum-deprived cells in a time-dependent manner, while the total BSG abundance did not change (Figure 5A). As a control for membrane and cytosolic proteins, the blot was probed with antibodies against p90RSK and EGFR. Immunofluorescence staining showed that subcellular distribution of BSG was observed predominantly at the membrane upon serum stimulation (Figure 5B). We next determined whether serum-induced BSG membrane recruitment could be affected by TRAF6. As shown in Figure 5C, BSG membrane recruitment was significantly decreased in TRAF6 knockdown cells, with little change in total BSG abundance. TRAF6 is a well-known E3 ubiquitin ligase that mediates K63 ubiquitination [11]. Unlike K48-linked ubiquitination usually causing protein degradation, K63-linked ubiquitination is usually involved in pathway activation and protein trafficking [11]. Since BSG is a novel interacting partner of TRAF6, we tested the possibility that TRAF6 might ubiquitinate BSG by co-transfecting TRAF6-WT or TRAF6-DN (C70A mutation, which abolishes TRAF6's E3 ligase activity) along with BSG and Ub-K63-HA plasmids into 293T cells. Compared with control and TRAF6-DN cells, there was an increase in the ubiquitination of BSG in cells expressing TRAF6-WT (Figure 5D). Interestingly, we also showed that BSG ubiquitination was induced by serum stimulation, as evidenced by the finding that the endogenous ubiquitination of BSG, detected with P4D1 antibody, in SK-MEL-5 cells was induced by15 min of serum stimulation (Figure 5E).

### Lysine residues at BSG cytoplasmic domain are responsible for TRAF6-mediating BSG ubiquitination

**Figure 6:**
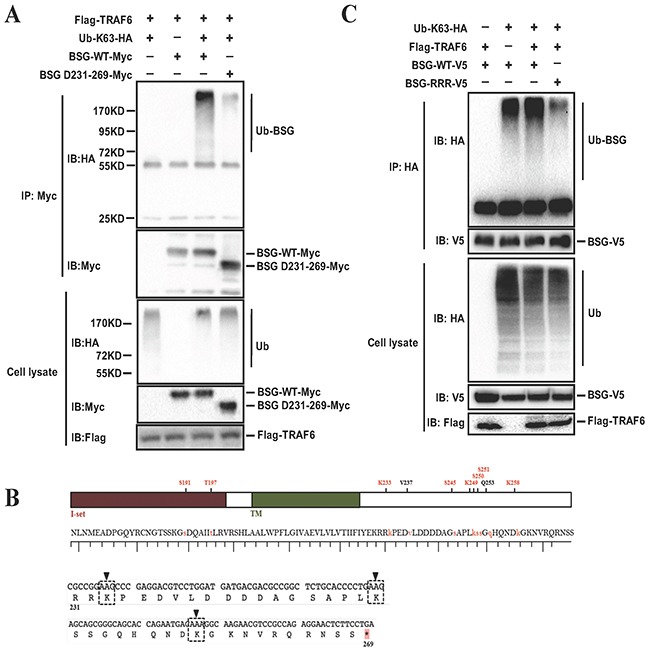
Lysine residues at BSG cytoplasmic domain are responsible for BSG ubiquitination mediated by TRAF6 **A.** Cytoplasmic domain of BSG is ubiquitinated by TRAF6. 293T cells were co-transfected with Flag-TRAF6 and BSG-Myc or BSG-D231-269-Myc, along with Ub-K63-HA. At 36 h post-transfection, cell lysates were immunoprecipitated with anti-Myc. **B.** Schematic diagram of BSG mutant constructs, in which all of the lysine residues at the cytoplasmic domain were replaced with arginine (BSG-RRR). **C.** BSG-RRR-V5 mutants and Flag-TRAF6, along with Ub-K63-HA were co-transfected into 293T cells, detection was performed as described above.

To determine which region of BSG is ubiquitinated by TRAF6, we compared full length BSG with a BSG deletion-mutant that lacks the cytoplasmic domain D231-269. K63-linked polyubiquitin was found to be significantly decreased in the BSG mutant (Figure 6A), suggesting that the intracellular domain of BSG is ubiquitinated by TRAF6. Examination of the database (http://www.phosphosite.org/proteinAction) an online resource that provides information on the post-translational modifications of proteins based on large-scale mass spectrometry data, revealed three lysine residues, Lys233, Lys249 and Lys258, at the cytoplasmic domain of BSG. We then constructed the BSG mutant (BSG-RRR) by replacing lysine residues with arginine, which renders BSG defective in ubiquitination (Figure 6B), showed impaired ubiquitination compared to the full-length BSG (Figure 6C), providing the evidence that TRAF6 ubiquitinates BSG at its cytoplasmic lysine residues.

### TRAF6 regulates MMP-9 expression through BSG

**Figure 7:**
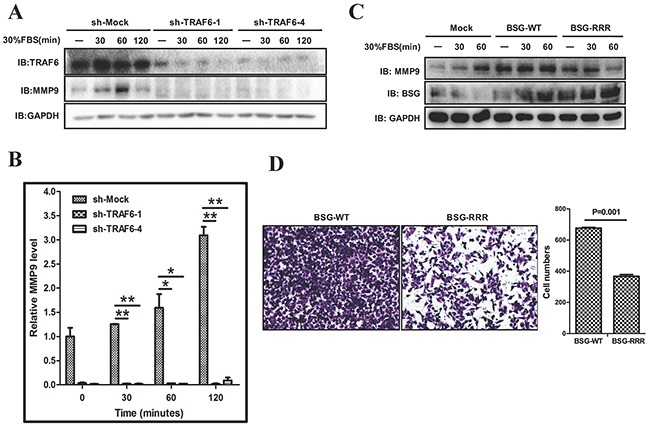
TRAF6-BSG axis is required for MMP9 induction **A.** and **B.** SK-MEL-5 cells expressing shMock and shTRAF6 (#1 and #4) were starved for 16 h. After stimulation with 30% FBS, cell lysates and mRNA were analyzed by immunoblotting (A), and qPCR (B). **C.** SK-MEL-5 cells were transfected with control, full length of BSG or BSG-RRR. At 36 h post-transfection, cells were starved for 16 h and stimulated with 30% FBS. Cell lysates were analyzed by immunoblotting with indicated antibodies. **D.** Transwell experiment was performed with SK-MEL-5 cells transfected with BSG-WT or BSG-RRR plasmids.

Matrix metalloproteinases (MMPs) play crucial roles in cancer cell invasion and metastasis by mediating extracellular matrix (ECM) degradation and remodeling [25], which leads to the breakdown of barriers for metastatic spread. BSG (CD147, EMMPRIN) is an inducer of tumor cell associated MMPs, including MMP1, MMP2, MMP3, and MMP9 [26–29]. Over-expression of MMP2 or MMP9 is often associated with melanoma metastasis and lesions [30–32]. In view of our data showing that TRAF6 contributes to melanoma metastasis *in vitro* and *in vivo*, we tested whether TRAF6 might regulate the expression of MMPs. Indeed, MMP9 expression was dramatically decreased in TRAF6 knockdown cells at both the mRNA and protein levels (Figure 7A, 7B). In addition, we examined the possible involvement of the ubiquitination sites of BSG in the regulation of MMP9 expression. In support of the notion that TRAF6-mediated BSG ubiquitination has important functional consequence, BSG-RRR exhibited a compromised ability to induce MMP9 expression (Figure 7C) and diminished cell invasiveness (Figure 7D).

## DISCUSSION

Accumulating evidences indicate that TRAF6 has oncogenic characteristics [12, 14, 33–35]. Our study uncovered previously unrecognized roles of TRAF6 in melanoma invasion and metastasis. We present evidence showing that TRAF6 is overexpressed in clinical melanoma tissues, including metastatic melanoma (Figure 1). The oncogenic function of TRAF6 in melanoma progression was demonstrated by the findings that knockdown of TRAF6 in melanoma cells resulted in diminished malignant phenotypes both *in vitro* and *in vivo* (Figures 2 and 3), suggesting that TRAF6 plays a critical role in melanoma metastasis. Interestingly, we found that TRAF6 interacts with BSG through directly binding to its transmembrane domain (Figure 4). BSG has been shown to promote invasion and metastasis by inducing the production and activity of MMPs [27–29, 36, 37]. In addition, BSG functions as a chaperone protein with other proteins to influence cell adhesion [38], glycolysis [19], angiogenesis [39], and chemoresistance [40].

As a glycosylated transmembrane protein, the *N*-glycosylation modification plays important role for BSG function. Although it was reported that only glycosylated BSG is able to stimulate MMPs production, mutation of glycosylation sites in BSG impairs its ability to induce MMPs expression [41]. However, post-translational modifications of BSG and its biological function remain largely unknown so far. Protein ubiquitination is an important post-translational modification that plays essential roles in regulating protein functions and thereby controlling numerous critical cellular processes. Ubiquitination can either mark proteins for degradation or activate signaling pathways [42, 43]. E3 ligases bind to their substrates directly and so have substrate specificity, are considered to be the most important component of the ubiquitin machinery. Their roles in cancer-related processes are now being discovered. TRAF6 was identified as an E3 ligase for Akt, with K63-linked polyUb regulating its membrane localization and phosphorylation, which are critical for Akt activation [11]. It was also reported that TRAF6 is recruited, and its E3 ligase activity is increased in response to EGFR activation, enhancing EGFR-mediated tumorigenesis [44].

In this study, we demonstrated that TRAF6 promotes K63-linked ubiquitination of BSG, which was induced by serum stimulation. Interestingly, TRAF6 seemed to induce BSG membrane recruitment via its E3 ligase activity as the E3 ligase-mutant failed to elicit such an effect (Figure 5). Of note is that TRAF6 targets the intracellular domain of BSG for ubiquitination. We identified three lysine residues in the intracellular domain of BSG as the acceptor sites for TRAF6-induced BSG ubiquitination (Figure 6). In addition, TRAF6-mediated K63-linked ubiquitination appeared to be important in the regulation of the activity of BSG-dependent MMP9 expression and strongly affects cell invasiveness (Figure 7). Both TRAF6 -knockdown and deletion of the Lys233, Lys249 and Lys258 residues in the cytoplasmic domain of BSG resulted in diminished expression of MMP9 and reduced cell invasion assay in a Matrigel transwell assay. Given the well-documented role of MMP9 in cancer metastasis and the direct effect of BSG ubiquitination on cell invasiveness in our present study, TRAF6-mediated BSG ubiquitination represents a novel mechanism underlying BSG-dependent melanoma metastasis. Further study is necessary to investigate how BSG ubiquitination can regulate its membrane distribution and what is the subsequent machinery of enriched membrane BSG in downstream signaling pathways.

In summary, we uncovered that TRAF6 is overexpressed in human melanoma tissues. Knock down of TRAF6 significantly attenuates malignant phenotypes including cell growth, migration, invasion and metastasis *in vitro* and *in vivo*. In particular, we demonstrated that BSG is a novel interacting partner of TRAF6, which regulates BSG membrane recruitment, BSG-dependent MMP9 expression and melanoma cells invasiveness via K63-linked ubiquitination. Our data implicate TRAF6 as a potential molecular target for chemotherapy and prevention against malignant melanoma.

## MATERIALS AND METHODS

### Reagents and antibodies

Chemical reagents, including Tris, NaCl, and SDS for molecular biology and buffer preparation were purchased from Sigma-Aldrich (St. Louis, MO). Cell culture media and other supplements were purchased from Life Technologies, Inc. (Rockville, MD). TRAF6 antibody (Santa Cruz, CA, USA) was diluted at 1:500, actin antibody (Santa Cruz, CA, USA) was diluted at 1:1000, GAPDH antibody (Proteintech, USA) was diluted at 1:8000, c-Myc antibody (Santa Cruz, CA, USA) was diluted at 1:1000, Flag antibody (Sigma, Germany) was diluted at 1:10000, CD147 antibody (Santa Cruz, CA, USA) was diluted at 1:500, MMP9 antibody (Abcam, Cambridge, UK) was diluted at 1:1000, HA antibody (Santa Cruz, CA, USA) was diluted at 1:500, P4D1 (Cell Signaling Technology, Danvers, MA) was diluted at 1:250, EGFR antibody, p90RSK antibody (Cell Signaling Technology, Danvers, MA) were both diluted at 1:1000.

### Construction of expression vectors

Expression constructs, including pRK5-HA-Lys-63-ubiquitin and pCDNA3.0-FlagTRAF6-WT were obtained from Addgene (Cambridge, MA) Lentivirus plasmids containing pLKO.1-shMock and pLKO.1-shTRAF6 (#1:TRCN0000007348, #3:TRCN0000007351, #4: TRCN0000007352) were purchased from Thermo Scientific (Huntsville, AL). The pCDNA4ToA-BSG-Myc and BSG deletion mutants were constructed or maintained in our lab previously [36]. pCDNA3.0-Flag-TRAF6-C70A was generated from TRAF6-WT using a site-directed mutagenesis kit (Strategene, La Jolla, CA).

### Cell culture, transfection and lentiviral infection

The human malignant melanoma cell lines SK-MEL-5 and SK-MEL-28 (American Type Culture Collection, Manassas VA, USA), and the 293T (maintained in our lab) cells were grown in Dulbecco's modified Eagle's medium (DMEM, Thermo Scientific, MA, USA) supplemented with 10% fetal bovine serum (FBS) and antibiotics.

For transfection experiments, cells were transfected with different plasmids using TurboFect Transfection Reagent (Thermo Scientific, MA, USA). The reagent and DNA were diluted in Opti-MEM (Invitrogen, CA, USA) and incubated for 15 min. The mixture was added to cells growing in the plates for 36 to 48 h to facilitate transfection. To generate stable TRAF6 knockdown cells, pLKO.1-shTRAF6 or pLKO.1-shMock plasmids were co-transfected with packaging plasmids (PSPAX2 and PMD2-G) into 293T cells. The supernatant fraction containing lentiviral particles was collected at 48 and 72 h separately, followed infection into SK-MEL-5 and SK-MEL-28 cells supplemented with 10 μg/mL polybrene. At 16 h after infection, the medium was replaced with fresh medium containing the suitable concentration of puromycin. The appropriate experiments were performed with these cells, until the control cells (without infection) completely died (usually 2-3 days) in the puromycin medium.

### Immunoblotting and immunoprecipitation

Cells were lysed in RIPA buffer and protein concentrations were determined by a BCA Protein Assay Kit (Santa Cruz, CA, USA). Immunoblotting were performed as described previously [22]. For immunoprecipitation, extracts were pre-cleared with 30 μL agarose A/G beads (Beyotime Institute of Biotechnology) by rotating for 1 h at 4°C. Beads were removed, and then another 30 μL agarose A/G beads and 1.5 μg antibodies were added to the lysates rotating overnight at 4°C. The beads were then washed twice in basic lysis buffer, and boiled for 10 min in loading buffer before resolving by SDS-PAGE. The blots were detected by an imaging system (Bio-Rad, USA).

### Immunofluorescence

SK-MEL-5 cells were grown on coverslips, starved overnight and stimulated with 30% FBS in DMEM. Cells were fixed in 4% paraformaldehyde for 10 min, blocked in 1% BSA for 30 min, and incubated with primary antibody overnight at 4°C. The treated cells were then incubated with fluorescent secondary antibody for 30 min in the dark and mounted with glycerol and observed with the Zeiss LSM 510 microscope (Carl Zeiss, Thornwood, NY).

### MTS assay

Cells were seeded (1×10^3^/well) in 96-well plates to allow attachment and incubated overnight. Cell proliferation was measured by MTS assay (Promega), according to the instruction. Each sample had 5 replicates.

### Anchorage-independent growth assay

Cells (8×10^3^ /well) were suspended in 1mL of Basal Medium Eagle (BME) supplemented with 10% FBS and 0.33% agar, and then seeded into 3 mL of solidified BME supplemented with 10% FBS and 0.5% agar in 6-well plates. Colonies were scored using a microscope and the Image J computer program. Statistical analyses were performed using Prism 5.0 statistics software.

### Tumor xenograft growth and lung metastasis mouse model

Xenografts tumor models were established by our lab before [16] and the animal study was approved by the Ethics Committee of Xiangya Hospital (Central South University, China). SK-MEL-5 cells transduced with sh-Mock, sh-TRAF6-1 or sh-TRAF6-4 lentiviral particles were collected, washed with PBS buffer, resuspended in cold serum-free DMEM, and subcutaneously injected (5×10^6^/0.15 mL) into the right flank of 4-6-week-old male BALB/c nude mice (Shanghai SLAC Laboratory Animal Co. Ltd., Shanghai, China). Tumors were measured twice a week using calipers and the tumor volumes were calculated using the formula: length × width × height × 0.52. In the lung metastasis experiment, suspended cells (2×10^6^ /0.15 mL) were injected into the lateral tail vein of 5-6-week-old male mice. Animals were sacrificed one month after tumor cell inoculation. Lung tissues were harvested and fixed in 10% buffered formalin, embedded in paraffin, sectioned at 5 μm, and stained with H&E or subjected to immunohistochemical analysis.

### Immunohistochemical analysis

A human melanoma tissue array (ME1004b) was purchased from Alenabio Biotechnology (Beijing, China). It included 56 primary melanomas, 23 metastatic melanomas and 18 normal nevi. The slide was stained according to the manufacturer's protocol. Briefly, the slide was baked at 60°C for 2 h, dewaxed in turpentine and rehydrated in a graded ethanol series, and then treated with 3% hydrogen peroxidefor 10 min to inhibit endogenous peroxidase. The slide was pretreated in a microwave oven (in 0.01 M sodium citrate buffer, pH 6.0) for 5 min, blocked with 10% goat serum for 30 min and then incubated with TRAF6 antibody (1:100, Santa Cruz, CA) at 4°C in a humidified chamber overnight. The slide was washed the next day and incubated with the secondary antibody (anti-rabbit 1:200, Santa Cruz, CA) for 1 h. Horseradish peroxidase-streptavidin (Santa Cruz, CA) was added to the slide. The samples were stained for 5 min with a 0.05% 3, 3′-diaminobenzidine substrate and counterstained with hematoxylin for 5 min and then mounted in neutral balsam.

### Immunohistochemical evaluation

Immunohistochemical staining were assessed independently by two investigators blinded to the identity of the tissue sections. Three fields were randomly selected under microscope at a magnification of 400×. Staining intensity of tissue sections was graded from 0 to 3: negative as 0; weakly positive as 1; moderately positive as 2; strongly positive as 3 while the proportion of immune-reactive cells was scored as follows: none: 0; 1-25%: 1; 26-50%: 2; more than 50%: 3. The immunoreactivity intensity distribution index (IRIDI) was calculated by multiplying the two scores. Samples that were unrecognizable or heavily covered by melanin were excluded. 34 primary melanomas arising from skin, 19 metastatic melanomas and 18 normal nevi were included in our final assessment.

### Preparation of membrane and cytosolic fractions

Cells were grown in 10-cm dishes and cultured to 70-80% confluence, starved for 16 h and then stimulated with 30% FBS and harvested at different time points. Membrane and cytosolic fractions were prepared using the Membrane Protein Extraction Reagent kit (Thermo Scientific, USA).

### Wound healing and transwell assay

For migration assay, transfected cells were plated at 4×10^5^/2 mL in 6-well plates. The medium was replaced by serum-free DMEM and wounds were made with a pipette tip after cells were attached. Images of the wound closure were photographed at different time points (0, 24, 48 and 72 h). For invasion assay, transwell experiment was performed with the 8μm-pore chamber inserted into 24-well plates (corning, NY, USA). Matrigel (BD) was diluted (1:7) in serum-free DMEM, and then added to each chamber and allowed to solidify completely. Transfected cells were obtained and resuspended in serum-free medium at a concentration of 5 × 10^4^/100 μL and seeded in the upper chambers while 600μL DMEM containing 30% FBS, used as a chemotactic factor, was placed at the bottom of the chamber. After 24h incubation, cells were fixed with 4% paraformaldehyde in PBS and stained with crystal violet for 20 min. The number of cells that migrated to the lower surface of the membrane were counted and images were taken using an inverted microscope.

### Quantitative real-time PCR analysis

Total RNA was extracted from cells infected with *sh-Mock*, *sh-TRAF4#1* or *4* after treatment with serum at various time points using the Qiagen RNeasy kit (Qiagen) according to the manufacturer's instructions. Total RNA (3 mg) was used as a template for the reverse transcription reaction (SuperScript III First-Strand Synthesis System for reverse transcription–PCR, Invitrogen). The *MMP-9* primers used were as follows Forward: 5-gaaccaatctcaccgacagg-3; Reverse 5-gccacccgagtgtaaccata-3.

### Statistical analysis

Data were expressed as mean ± SEM. Student's *t* test or one-way ANOVA was used to determine the statistical differences. A p value of less than 0.05 was considered statistically significant.

## References

[1] Trinh VA (2008). Current management of metastatic melanoma. Am J Health Syst Pharm.

[2] Simard EP, Ward EM, Siegel R, Jemal A (2012). Cancers with increasing incidence trends in the United States: 1999 through 2008. CA Cancer J Clin.

[3] (2014). Cancer Facts & Figures 2014.

[4] Arch RH, Gedrich RW, Thompson CB (1998). Tumor necrosis factor receptor-associated factors (TRAFs)---a family of adapter proteins that regulates life and death. Genes & Development.

[5] Yin Q, Lin SC, Lamothe B, Lu M, Lo YC, Hura G, Zheng L, Rich RL, Campos AD, Myszka DG, Lenardo MJ, Darnay BG, Wu H (2009). E2 interaction and dimerization in the crystal structure of TRAF6. Nat Struct Mol Biol.

[6] Takeuchi M, Rothe M, Goeddel DV (1996). Anatomy of TRAF2: Distinct domains for nuclear factor- b activation and association with tumor necrosis factor signaling proteins. Journal of Biological Chemistry.

[7] Lorick KL, Jensen JP, Fang S, Ong AM, Hatakeyama S, Weissman AM (1999). RING fingers mediate ubiquitin-conjugating enzyme (E2)-dependent ubiquitination. Proc Natl Acad Sci U S A.

[8] Cao Z, Xiong J, Takeuchi M, Kurama T, Goeddel DV (1996). TRAF6 is a signal transducer for interleukin-1. Nature.

[9] Deng L, Wang C, Spencer E, Yang L, Braun A, You J, Slaughter C, Pickart C, Chen ZJ (2000). Activation of the IkappaB kinase complex by TRAF6 requires a dimeric ubiquitin-conjugating enzyme complex and a unique polyubiquitin chain. Cell.

[10] Wang C, Deng L, Hong M, Akkaraju GR, Inoue J, Chen ZJ (2001). TAK1 is a ubiquitin-dependent kinase of MKK and IKK. Nature.

[11] Yang WL, Wang J, Chan CH, Lee SW, Campos AD, Lamothe B, Hur L, Grabiner BC, Lin X, Darnay BG, Lin HK (2009). The E3 ligase TRAF6 regulates Akt ubiquitination and activation. Science.

[12] Starczynowski DT, Lockwood WW, Delehouzee S, Chari R, Wegrzyn J, Fuller M, Tsao MS, Lam S, Gazdar AF, Lam WL, Karsan A (2011). TRAF6 is an amplified oncogene bridging the RAS and NF-kappaB pathways in human lung cancer. J Clin Invest.

[13] Starczynowski DT, Kuchenbauer F, Argiropoulos B, Sung S, Morin R, Muranyi A, Hirst M, Hogge D, Marra M, Wells RA, Buckstein R, Lam W, Humphries RK, Karsan A (2010). Identification of miR-145 and miR-146a as mediators of the 5q- syndrome phenotype. Nat Med.

[14] Sun H, Li XB, Meng Y, Fan L, Li M, Fang J (2013). TRAF6 upregulates expression of HIF-1alpha and promotes tumor angiogenesis. Cancer Res.

[15] Liu J, Xu J, Li H, Sun C, Yu L, Li Y, Shi C, Zhou X, Bian X, Ping Y, Wen Y, Zhao S, Xu H, Ren L, An T, Wang Q (2015). miR-146b-5p functions as a tumor suppressor by targeting TRAF6 and predicts the prognosis of human gliomas. Oncotarget.

[16] Chen X, Lin J, Kanekura T, Su J, Lin W, Xie H, Wu Y, Li J, Chen M, Chang J (2006). A small interfering CD147-targeting RNA inhibited the proliferation, invasiveness, and metastatic activity of malignant melanoma. Cancer Res.

[17] Arendt BK, Walters DK, Wu X, Tschumper RC, Huddleston PM, Henderson KJ, Dispenzieri A, Jelinek DF (2012). Increased expression of extracellular matrix metalloproteinase inducer (CD147) in multiple myeloma: role in regulation of myeloma cell proliferation. Leukemia.

[18] Ke X, Fei F, Chen Y, Xu L, Zhang Z, Huang Q, Zhang H, Yang H, Chen Z, Xing J (2012). Hypoxia upregulates CD147 through a combined effect of HIF-1alpha and Sp1 to promote glycolysis and tumor progression in epithelial solid tumors. Carcinogenesis.

[19] Su J, Chen X, Kanekura T (2009). A CD147-targeting siRNA inhibits the proliferation, invasiveness, and VEGF production of human malignant melanoma cells by down-regulating glycolysis. Cancer Lett.

[20] Gabison EE, Hoang-Xuan T, Mauviel A, Menashi S (2005). EMMPRIN/CD147, an MMP modulator in cancer, development and tissue repair. Biochimie.

[21] Sun J, Hemler ME (2001). Regulation of MMP-1 and MMP-2 production through CD147/extracellular matrix metalloproteinase inducer interactions. Cancer Res.

[22] Luo Z, Zeng W, Tang W, Long T, Zhang J, Xie X, Kuang Y, Chen M, Su J, Chen X (2014). CD147 interacts with NDUFS6 in regulating mitochondrial complex I activity and the mitochondrial apoptotic pathway in human malignant melanoma cells. Curr Mol Med.

[23] Stenzinger A, Wittschieber D, von Winterfeld M, Goeppert B, Kamphues C, Weichert W, Dietel M, Rabien A, Klauschen F (2012). High extracellular matrix metalloproteinase inducer/CD147 expression is strongly and independently associated with poor prognosis in colorectal cancer. Hum Pathol.

[24] Zhao S, Ma W, Zhang M, Tang D, Shi Q, Xu S, Zhang X, Liu Y, Song Y, Liu L, Zhang Q (2013). High expression of CD147 and MMP-9 is correlated with poor prognosis of triple-negative breast cancer (TNBC) patients. Med Oncol.

[25] Liotta LA, Tryggvason K, Garbisa S, Hart I, Foltz CM, Shafie S (1980). Metastatic potential correlates with enzymatic degradation of basement membrane collagen. Nature.

[26] Biswas C, Zhang Y, DeCastro R, Guo H, Nakamura T, Kataoka H, Nabeshima K (1995). The human tumor cell-derived collagenase stimulatory factor (renamed EMMPRIN) is a member of the immunoglobulin superfamily. Cancer Res.

[27] Caudroy S, Polette M, Nawrocki-Raby B, Cao J, Toole BP, Zucker S, Birembaut P (2002). EMMPRIN-mediated MMP regulation in tumor and endothelial cells. Clin Exp Metastasis.

[28] Kanekura T, Chen X, Kanzaki T (2002). Basigin (CD147) is expressed on melanoma cells and induces tumor cell invasion by stimulating production of matrix metalloproteinases by fibroblasts. Int J Cancer.

[29] Tang Y, Kesavan P, Nakada MT, Yan L (2004). Tumor-stroma interaction: positive feedback regulation of extracellular matrix metalloproteinase inducer (EMMPRIN) expression and matrix metalloproteinase-dependent generation of soluble EMMPRIN. Mol Cancer Res.

[30] van den Oord JJ, Paemen L, Opdenakker G, de Wolf-Peeters C (1997). Expression of gelatinase B and the extracellular matrix metalloproteinase inducer EMMPRIN in benign and malignant pigment cell lesions of the skin. Am J Pathol.

[31] MacDougall JR, Matrisian LM (1995). Contributions of tumor and stromal matrix metalloproteinases to tumor progression, invasion and metastasis. Cancer Metastasis Rev.

[32] Vaisanen A, Tuominen H, Kallioinen M, Turpeenniemi-Hujanen T (1996). Matrix metalloproteinase-2 (72 kD type IV collagenase) expression occurs in the early stage of human melanocytic tumour progression and may have prognostic value. J Pathol.

[33] Beroukhim R, Mermel CH, Porter D, Wei G, Raychaudhuri S, Donovan J, Barretina J, Boehm JS, Dobson J, Urashima M, Mc Henry KT, Pinchback RM, Ligon AH, Cho YJ, Haery L, Greulich H (2010). The landscape of somatic copy-number alteration across human cancers. Nature.

[34] Meng Q, Zheng M, Liu H, Song C, Zhang W, Yan J, Qin L, Liu X (2012). TRAF6 regulates proliferation, apoptosis, and invasion of osteosarcoma cell. Mol Cell Biochem.

[35] Sun H, Li X, Fan L, Wu G, Li M, Fang J (2014). TRAF6 is upregulated in colon cancer and promotes proliferation of colon cancer cells. Int J Biochem Cell Biol.

[36] Long T, Su J, Tang W, Luo Z, Liu S, Liu Z, Zhou H, Qi M, Zeng W, Zhang J, Chen X (2013). A novel interaction between calcium-modulating cyclophilin ligand and Basigin regulates calcium signaling and matrix metalloproteinase activities in human melanoma cells. Cancer Lett.

[37] Zeng W, Su J, Wu L, Yang D, Long T, Li D, Kuang Y, Li J, Qi M, Zhang J, Chen X (2014). CD147 promotes melanoma progression through hypoxia-induced MMP2 activation. Curr Mol Med.

[38] Li Y, Wu J, Song F, Tang J, Wang SJ, Yu XL, Chen ZN, Jiang JL (2012). Extracellular membrane-proximal domain of HAb18G/CD147 binds to metal ion-dependent adhesion site (MIDAS) motif of integrin beta1 to modulate malignant properties of hepatoma cells. J Biol Chem.

[39] Voigt H, Vetter-Kauczok CS, Schrama D, Hofmann UB, Becker JC, Houben R (2009). CD147 impacts angiogenesis and metastasis formation. Cancer Invest.

[40] Kuang YH, Chen X, Su J, Wu LS, Liao LQ, Li D, Chen ZS, Kanekura T (2009). RNA interference targeting the CD147 induces apoptosis of multi-drug resistant cancer cells related to XIAP depletion. Cancer Lett.

[41] Papadimitropoulou A, Mamalaki A (2013). The glycosylated IgII extracellular domain of EMMPRIN is implicated in the induction of MMP-2. Mol Cell Biochem.

[42] Chen ZJ, Sun LJ (2009). Nonproteolytic functions of ubiquitin in cell signaling. Mol Cell.

[43] Ciechanover A (2005). Proteolysis: from the lysosome to ubiquitin and the proteasome. Nat Rev Mol Cell Biol.

[44] Feng H, Lopez GY, Kim CK, Alvarez A, Duncan CG, Nishikawa R, Nagane M, Su AJ, Auron PE, Hedberg ML, Wang L, Raizer JJ, Kessler JA, Parsa AT, Gao WQ, Kim SH (2014). EGFR phosphorylation of DCBLD2 recruits TRAF6 and stimulates AKT-promoted tumorigenesis. J Clin Invest.

